# Frailty is associated with poor sleep quality in the oldest old

**DOI:** 10.3906/sag-2001-168

**Published:** 2021-04-30

**Authors:** Çağatay ÇAVUŞOĞLU, Olgun DENİZ, Rana Tuna DOĞRUL, Süheyla ÇÖTELİ, Ali ÖNCÜL, Muhammet Cemal KIZILARSLANOĞLU, Berna GÖKER

**Affiliations:** 1 Division of Geriatric Medicine, Department of Internal Medical Sciences, Faculty of Medicine, Gazi University, Ankara Turkey; 2 Division of Geriatrics and Palliative Care, Department of Internal Medical Sciences, University of Health Sciences,Konya Education and Research Hospital, Konya Turkey

**Keywords:** Frailty, insomnia, oldest old, sleep disorders, sleep quality

## Abstract

**Background/aim:**

Sleep disorders and frailty increase with advancing age, along with physical disabilities, cognitive dysfunction, mood disorders, and social vulnerability. Thus, the study objective was to evaluate the relationship between frailty and sleep quality in the oldest old patients.

**Materials and methods:**

In this study, 100 patients aged ≥80 years were assessed using comprehensive geriatric assessment (CGA) including basic activities of daily living (ADL), instrumental ADL, handgrip strength, the Geriatric Depression Scale-15, the Mini-Mental State Examination, and the Mini-Nutritional Assessment-Short Form. The sleep quality and frailty status of the patients were evaluated using the Pittsburgh Sleep Quality Index (PSQI) and the Fried Frailty Index, respectively.

**Results:**

The median age of the participants was 84 years (80–92), 55% of them were women, and 41% of them were frail. There was no statistically significant difference between the frail and nonfrail groups in terms of age, sex, and comorbidities (P > 0.050). The frail patients scored poorly according to the CGA tests when compared to the nonfrail ones (P < 0.050). The median score for the PSQI was significantly higher in the frail group, 12 points (3–19) versus 6 points (1–19) in the nonfrail patients (P < 0.001). The PSQI score (odds ratio [OR] of 1.308, 95% confidence interval [CI]: 1.092–1.566, P = 0.004), female sex (OR of 5.489, 95% CI: 1.063–28.337; P = 0.042), and the basic ADL score (OR of 0.383; 95% CI: 0.207–0.706; P = 0.002) were found to be independently associated with frailty using multivariate analysis.

**Conclusion:**

Sleep quality was significantly decreased in the oldest old frail patients compared to the nonfrail ones, and poor sleep quality was independently associated with frailty. Evaluating the sleep patterns of the oldest old patients with CGA in daily geriatric practice might help to improve the quality of life of frail patients.

## 1. Introduction

Epidemiological studies have shown that more than 50% of people aged ≥65 years have sleep disorders [1–5], the incidence of which increases with advancing age [6–8]. Poor sleep quality and sleep disorders are associated with decreased cognitive function, escalated falling, worsening health status, and increased mortality [6]. More than half the participants in an epidemiological study on 9000 people aged ≥65 years, which was carried out at three different centers, reported having chronic sleep-related problems (i.e. trouble falling asleep, wakening during the night, wakening too early, difficulties with initiating or maintaining sleep, and insomnia) [9,10].

Frailty is a geriatric syndrome, and frail older adults are at risk of increased physical and cognitive vulnerability, as well as mood and social vulnerability. Frailty contributes to disease prognosis and negatively impacts mortality and morbidity. Impaired responses and increased sensitivity to external stressors contribute to frailty. Frailty is characterized by negative changes in the physiological capacity of multiple organs [11]. A decrease in psychological and cognitive functions negatively affects individuals socially. Various factors, including the environment, the frequency of daily communication with other people, and the ability to exercise and go out, significantly contribute to the health of older adults. If their needs are not met, they can be at risk of social vulnerability [12]. Makizako et al. defined social vulnerability as spending less time with friends, a reduction in communications with others, and decreased self-efficacy; in turn, social vulnerability leads to an increase in the levels of addiction and disabilities in older adults [13]. Frailty can result in an increase in the number of falls, deterioration in general health status, and premature death in older adults, and it is closely associated with impacted sleep parameters, such as deterioration in sleep quality, difficulty falling asleep, and distortion in the sleep–wake cycle [6,14]. The prevalence of frailty increases with advancing age. For example, it has a prevalence rate of 7% for those aged 65–74 years, while the rate increases to 40% in those aged ≥85 years [6,14]. Sleep disturbances adversely affect the general health of older populations and increase in frequency with advancing age [7,14]. Many studies have demonstrated that the oldest old individuals (aged ≥80 years) experience a greater number of physical, mental, and social changes than their younger counterparts (those aged 60–79 years) [15,16].

The relationship between frailty and sleep quality has not been adequately studied before, in particular with regard to the oldest old patients. Thus, the study objective was to determine the relationship between frailty and sleep quality in these oldest old patients.

## 2. Methods

### 2.1. Sample and study design

The current study was conducted between January 2019 and April 2019. The study included 100 consecutively presenting oldest old patients aged ≥80 years admitted to the geriatric outpatient clinic at Gazi University Hospital. A number of assessments were administered to the study participants during face-to-face interviews with a geriatrician. Cognitive function was assessed through a medical examination and comprehensive geriatric assessment (CGA) during an outpatient clinic visit. Patients who met the Petersen criteria for mild cognitive impairment or the Diagnostic and Statistical Manual of Mental Disorders-Fifth Edition (DSM-5) criteria for dementia were excluded from the study. We excluded all the patients with major depressive disorder and bipolar disorder according to the DSM 5 criteria. In addition, the patients with disabilities (amputations, stroke-induced sequela, aphasia, and hearing problems), decompensated heart failure, acute myocardial infarction, acute stroke, exacerbation of chronic obstructive pulmonary disease, acute illnesses (i.e. infections and unstable general conditions), and those admitted to hospital or the intensive care unit within the last 3 months were excluded. Written informed consent was obtained from the participants. The sociodemographic data of the patients, their educational status, and the presence of chronic comorbidities and medications were recorded. The CGA, sleep quality, and frailty evaluations were performed by the same geriatrician. The CGA, a multidisciplinary approach that determines the medical, psychosocial, and functional status of older adults, enables physicians to make diagnoses and develop treatment plans to minimize complications and side effects. In the current study, the basic activities of daily living (ADL), the instrumental activities of daily living (IADL), nutrition, cognition, mood, comorbidities, the number of medications, urinary incontinence, the number of falls within the last year, and fractures were included in the CGA [17]. The Pittsburgh Sleep Quality Index (PSQI) and the Fried Frailty Index (FFI) were used to evaluate sleep quality and frailty, respectively.

This study was performed in accordance with the Declaration of Helsinki and received the approval of the Gazi University Faculty of Medicine clinical research ethics committee (Reference number: 952).

### 2.2. Sleep parameters

A validated Turkish version of the PSQI, which comprises 24 questions, was used to assess the patients’ sleep quality [18,19]. Among these questions, nineteen were answered by the patients, and the remaining five questions were answered by each patient’s partner (who slept in the same room). However, the answers given by the partners were not included in the scoring. When evaluating the results, eight components were taken into consideration: (1) global score, (2) subjective sleep quality, (3) sleep latency, (4) sleep duration, (5) habitual sleep efficiency, (6) sleep disturbances, (7) the use of sleep medication, and (8) daytime dysfunction. The total score was obtained from a summation of the scores from all the components. The PSQI score ranged from 0 to 21 points, with high-end scores reflecting poor sleep quality [18,19]. A PSQI score of ≥5 indicated impaired sleep quality [19].

### 2.3. Frailty

Frailty is a geriatric syndrome and its prevalence increases with advancing age [7]. The FFI, developed by Fried et al. [14], is one of the most frequently used frailty indexes in clinical practice. It comprises five parameters of weight loss, exhaustion, low physical activity, slowness, and weakness. These factors are assessed and scored, with a maximum possible score of 5. The scores of 0, 1–2, and ≥3 points indicate that a person is robust, prefrail, and frail, respectively [14].

In the Fried criteria, the weight loss criterion means a loss of 4.5 or more kg within the last year. Patients with a Center for Epidemiological Studies Depression Scale score (CES-D) of ≥2 are considered to have exhaustion. A point is also added for physical activity performed by a man who consumes less than 383 kcal/week and physical activity carried out by a woman who consumes less than 270 kcal/week. The patient’s walking time over a distance of 4.6 km, adjusted for sex and height, is then determined. Finally, handgrip strength is assessed, and the results are interpreted based on the body mass index [14].

### 2.4. Other parameters

The sociodemographic data of the patients were evaluated, and the CGA was performed. Basic ADL scores were evaluated within the scope of the CGA [20,21]. A validated Turkish version of the basic ADL was used (scores of 0–6) [20,22]. The IADL scale (scores of 0–8) was also applied [23]. A validated Turkish version of the Mini-Mental State Examination was performed to evaluate the cognitive function (scores of 0–30) [24,25]. The Mini-Nutritional Assessment-Short-Form (MNA-SF) test was employed to assess the malnutrition risk (scores of 0–14) [26]. The MNA-SF is valid and reliable in the Turkish population [27]. The Geriatric Depression Scale-15 (GDS-15), developed by Yesavage, was utilized to evaluate the patients’ depression status. A validated Turkish version of the GDS-15 was also used [28]. The total test score can range from 0 to 15 points [28,29] and the patients were assessed for urinary incontinence. The number of falls within the last year, fractures, bedsores, comorbidities, and the number of medications used were also recorded.

### 2.5. Statistical analysis

The statistical analysis was performed with SPSS Statistics for Windows 22.0. The categorical parameters were expressed as number ( *n* ) and percentage (%). Whether the numerical parameters had a normal distribution was determined using a histogram, variation coefficients, and the Kolmogorov–Smirnov test. Normally distributed numerical parameters were expressed as mean ± deviation, and nonnormally distributed numerical data were expressed as median (minimum–maximum). Student’s *t* -test was utilized to compare the normally distributed numerical parameters between two independent groups, and the Mann–Whitney U test was used to compare the nonnormally distributed parameters. The categorical variables were compared using the chi-square or Fisher’s exact tests.

The patients were divided into two groups based on their frailty status (frail and nonfrail). First, we performed univariate analysis to detect the parameters related with frailty. The parameters that have a P-value of <0.200 were included in the multivariate analysis to identify the factors that were independently associated with frailty. When considering type one error as 0.05 and power as 0.80 to find out significant differences between frail and nonfrail oldest old patients regarding poor sleep quality, the minimum number of the patients included in the study is calculated as 74 (37 in the frail and 37 in the nonfrail groups). A P-value of <0.05 was considered statistically significant.

## 3. Results

This study included 100 patients. There were 41 frail and 59 nonfrail patients. Table 1 depicts the clinical characteristics of the participants. A statistically significant difference was not demonstrated between the frail and nonfrail groups in terms of age, sex, and comorbidities (P > 0.050). Age did not differ between the frail and nonfrail groups [82 years (80–92) vs. 84 years (80–91), respectively; P = 0.151]. The differences between the groups in terms of comorbidities such as diabetes, hypertension, smoking, chronic obstructive pulmonary disease, coronary artery disease, malignancy, and depression were not statistically significant (P > 0.050). Frail patients were more likely to have urinary incontinence than nonfrail patients (74.3% vs. 35.2%, respectively) (P < 0.001). Table 2 shows the findings of the CGA parameters in detail. The basic ADL, MMSE, and MNA-SF test scores were significantly lower and the GDS scores were higher in the frail group compared to the nonfrail group.

**Table 1:** General characteristics and comorbidities of the patients according to frailty status .

Properties	Not frail(n = 59)	Frailn = 41)	P-value
Sex (female), n (%)	28 (47.5)	27 (65.9)	0.069
Age (years), median (minimum–maximum)	84 (80–91)	82 (80–92)	0.151
Smoking, n (%)	2 (3.6)	0 (0.0)	0.235
Diabetes mellitus, n (%)	17 (30.4)	13 (33.3)	0.759
Hypertension, n (%)	40 (72.7)	33 (86.8)	0.103
Chronic obstructive pulmonary disease, n (%)	4 (7.3)	4 (10.8)	0.710
Osteoporosis, n (%)	6 (12.0)	10 (28.6)	0.054
Coronary artery disease, n (%)	15 (26.8)	13 (35.1)	0.390
Urinary incontinence, n (%)	19 (35.2)	26 (74.3)	< 0.001*

*: statistically significant.

**Table 2:** Comprehensive geriatric assessment tests’ results according to frailty status.

Parameters	Not frail(n = 59)	Frailn = 41)	P-value
Number of drugs	5 (1–14)	5 (0–19)	0.331
Number of comorbidities	3 (1–6)	3 (2–7)	0.028*
Basic ADL	6 (0–6)	4 (0–6)	< 0.001*
Instrumental ADL	7 (0–8)	4 (0–8)	< 0.001*
Mini-Mental State Examination	27 (0–30)	21 (7–30)	0.005*
Mini Nutritional Assessment	12 (3–14)	10.5 (4–14)	0.002*
Geriatric Depression Scale-15	2 (0–14)	6 (0–11)	0.001*

*: statistically significant, ADL: activities of daily living. The numerical parameters are presented as median (minimum–maximum).

Table 3 shows the distribution of the total PSQI scores and subscores by groups. The total PSQI scores and the subparameter scores (subjective sleep quality, sleep latency, sleep duration, habitual sleep efficiency, sleep disturbances, and daytime dysfunction) were found to be significantly lower in the nonfrail group compared to the frail group (P < 0.001). The difference between the groups in terms of the PSQI subscale, which is the use of sleep medications, was not statistically significant. Additionally, only 17 patients out of 100 in our study stated that they used sleeping drugs in the last month, and 11 of them stated that they used drugs 3 or more days a week. Only 6 of 11 patients used medication regularly. Of these patients, four used trazodone 50 mg daily and two of them used hydroxyzine hydrochloride 25 mg each day. There was no statistically significant difference about the use of sleeping drugs between the frail and nonfrail groups.

**Table 3:** Pittsburg Sleep Quality Index total and subgroup scores according to frailty status.

Parameters	Not frail(n = 59)	Frailn = 41)	P-value
PSQI total score	6 (1–19)	12 (3–19)	< 0.001*
Subjective sleep quality	1 (0–3)	2 (0–3)	< 0.001*
Sleep latency	1 (0–3)	2 (0–3)	0.003*
Sleep duration	0 (0–3)	2 (0–3)	0.039*
Habitual sleep efficiency	1 (0–3)	3 (0–3)	0.004*
Sleep disturbances	1 (0–3)	2 (1–3)	< 0.001*
Use of sleep medication	0 (0–3)	0 (0–3)	0.306
Daytime dysfunction	0 (0–3)	2 (0–4)	< 0.001*

*: statistically significant, PSQI: Pittsburg Sleep Quality Index. The numerical parameters are presented as median (minimum–maximum).

The PSQI, basic ADL, MMSE, MNA-SF, and GDS-15 scores, the number of comorbidities, sex, age, hypertension, and osteoporosis were included in the binary logistic regression model. The results of the logistic regression analysis are summarized in Table 4.

**Table 4:** Independently associated factors with frailty using multivariate analysis.

Parameters	Odds ratio	95% confidence interval		P-value
		Lower limit	Upper limit	
PSQI	1.308	1.092	1.566	0.004*
Basic ADL	0.383	0.207	0.706	0.002*
Sex (female)	5.489	1.063	28.337	0.042*

*: statistically significant, ADL: activities of daily living, PSQI: Pittsburg Sleep Quality Index. The parameters with a P-value of <0.200 were included in the multivariate analysis to identify factors that were independently associated with frailty. These parameters were PSQI, numbers of comorbidities, basic ADL, Mini-Mental State Examination, Mini-Nutritional Assessment, Geriatric Depression Scale-15, sex, age, hypertension, and osteoporosis. Backward stepwise model was used and last model (step-8) was presented in this table. Omnibus test for this model had a P-value of <0.001. Hosmer and Leme show test had a P-value of >0.050. Nagelkerke R square was 0.772 for this model.

## 4. Discussion

An independent relationship was demonstrated between frailty and sleep quality in the oldest old patients in the current study. In general, the prevalence of frailty and sleep problems is high in older adults, increasing with advancing age. Many systems are adversely affected by deterioration in sleep quality and frailty. It has been consistently reported in large epidemiological studies that frailty increases with advancing age, with the highest prevalence being reported in individuals aged 80 years [6]. For this reason, older adults aged ≥80 were included in the current study. The quality of life of the patients is adversely and significantly affected by frailty. A limited number of studies in the literature have explored the correlation between sleep quality and frailty in the oldest old patients. The present study demonstrated that the frailer the patients were, the more their sleep quality was impacted. Our findings are supported by those of a cohort study conducted on older, rural Mexican adults in which sleep complaints were associated with frailty in older women [29,30].

Frailty is an important geriatric syndrome, and it adversely affects geriatric test results. It has been shown that it leads to increased dependence and falls in older individuals [10,14]. Fried et al. also showed that cognitive scores were lower and depressive symptoms were higher in frail patients [14]. The findings in our study were similar to those of these studies. We established that frail patients were more likely to have urinary incontinence. Similarly, Berardelli et al. demonstrated a strong correlation between frailty and urinary incontinence. They also suggested that urinary incontinence is a key component of frailty [31].

When the relationship between frailty and sleep quality and the irrespective subparameters were examined, a significant relationship was established in all the instances, except the number of sleep medications. The participants in the frail group experienced reduced subjective sleep quality, poorer sleep latency and duration, greater disturbances in sleep habits, and increased daytime sleepiness. A significant relationship was observed concerning the total PSQI score between subjective sleep quality, sleep disturbances, and daytime dysfunction in the frail group (P < 0.001). We found a significant relationship between frailty and daytime sleepiness and decrease in sleep duration in a previous study [10]. The use of sleep medication was the only subparameter that did not have any relationship with frailty. Most of the patients in the current study did not use sleep medication. This may be related to the fact that the majority of the patients included in the study did not have the diagnosis of insomnia. Therefore, the medications used in the treatment of insomnia were not different between the study groups. On the other hand, one of the reasons why the study groups did not have significantly different frequencies in terms of using sleep-related medications may depend on the fact that the number of the patients taking these sleep-related medications was small.

The PSQI score, the ADL score, and female sex were independently associated with frailty according to the multivariate analysis. Multiple mechanisms are responsible for the independent relationship between the PSQI and frailty. Deterioration in sleep quality may indicate a worsening health status, increased depressive symptoms, and decreased physical activity [2,3,10,28]. Besides, immunological mechanisms, such as an increase in proinflammatory cytokines, deterioration in renal function, and an increase in chronic inflammation markers, relate to frailty [28]. Therefore, there might be a bidirectional relationship between frailty and sleep quality, resulting in a vicious cycle. The loss of basic ADL renders the oldest old patients more vulnerable and more physically dependent, and places them at a higher risk of poorer health than their (relatively) younger counterparts [15]. Therefore, the loss of basic ADL negatively impacts the frailty status of the oldest old patients.

Female sex was found to have an independent relationship with frailty in the current study. This finding was an expected outcome since it has been reported elsewhere that frailty is common in older women. In addition, women have lower muscle strength and leaner muscle mass compared to men, which may be another reason for the higher rates of frailty in women [7].

The small sample size was a limitation of our study. However, the study had adequate statistical power in terms of the number of participants included. The strength of our study was that only the oldest old (≥80 years) were recruited. The subjective assessment of sleep quality using the PSQI was another weakness. Polysomnography could have been performed to evaluate sleep disturbances objectively. However, it is neither a widely used nor readily available test. Further studies that assess insomnia using polysomnography and further evaluations of this study population are warranted. When using the PSQI, an increase in sleep duration positively influences the score. It has been reported elsewhere that an increase in sleep duration is associated with frailty [6]. Therefore, further studies are required to determine the optimum sleep duration in older adults.

The key strength of our research was that it is the first study to have evaluated the association between frailty and sleep quality in the oldest old. The fact that the oldest old patients are frailer than older adults indicates that many challenges, such as sleep disorders, should be examined in more detail in this population. Deterioration in sleep quality is a significant problem as it affects multiple systems and is often overlooked by physicians.

The finding that sleep quality was associated with frailty in the current study suggests that it is important to evaluate sleep disorders while performing the CGA, especially in the oldest old. Further studies are needed to elucidate whether sleep disturbances play a role in the development of frailty and whether treating them would lead to an improvement in frailty status.
